# Metabolic interactions between coral animal and endolithic bacterial communities

**DOI:** 10.1093/ismeco/ycaf193

**Published:** 2024-10-28

**Authors:** Po-Shun Chuang, Ting-Chang Hsu, Chih-Ying Lu, Sheng-Ping Yu, Po-Yu Liu, Sim Lin Lim, Yu-Hsiang Chen, Yu-Jing Chiou, Shan-Hua Yang, Pei-Ling Wang, Sen-Lin Tang

**Affiliations:** Biodiversity Research Center, Academia Sinica, Taipei City 11529, Taiwan; Biodiversity Research Center, Academia Sinica, Taipei City 11529, Taiwan; Institute of Marine Environment and Ecology, National Taiwan Ocean University, Keelung City 20224, Taiwan; Precious Instrument Center of New Ocean Researcher 2, National Taiwan Ocean University, Keelung City 20224, Taiwan; Biodiversity Research Center, Academia Sinica, Taipei City 11529, Taiwan; Molecular and Biological Agricultural Sciences Program Taiwan International Graduate Program, National Chung Hsing University and Academia Sinica, Taipei City 11529, Taiwan; Graduate Institute of Biotechnology, National Chung Hsing University, Taichung City 40227, Taiwan; Biodiversity Research Center, Academia Sinica, Taipei City 11529, Taiwan; Biodiversity Research Center, Academia Sinica, Taipei City 11529, Taiwan; School of Medicine College of Medicine, National Sun Yat-sen University, Kaohsiung City 804201, Taiwan; Biodiversity Research Center, Academia Sinica, Taipei City 11529, Taiwan; Biodiversity Research Center, Academia Sinica, Taipei City 11529, Taiwan; Biodiversity Research Center, Academia Sinica, Taipei City 11529, Taiwan; Institute of Fisheries Science, National Taiwan University, Taipei City 11529, Taiwan; Institute of Oceanography, National Taiwan University, Taipei City 11529, Taiwan; Biodiversity Research Center, Academia Sinica, Taipei City 11529, Taiwan

**Keywords:** Endoliths, Isopora palifera, metabolomics, nitrogen budget, organic matter, translocation

## Abstract

Coral skeletons constitute sources of nutrients and energy for holobiont. Although bacteria predominate in endolithic microbiomes of corals, their ecological functions have long been masked by those of symbiotic microalgae. In the skeleton of *Isopora palifera*, previous studies showed the absence of microalgae and a green layer dominated by green sulfur bacteria. This system, which excludes a contribution from microalgae, provides a perfect model for studying the role of endolithic bacteria in corals. Using this model, we examined the metabolite profile and translocation of organic matter between coral tissue and skeleton. Chromatography-time-of-flight-mass spectrometry and ultra-high-performance liquid chromatography tandem mass spectrometry revealed distinct metabolic profiles in tissue and different skeletal layers. A stable isotope incubation experiment further demonstrated ^13^C translocation between tissue and the green layer, but no translocation of ^15^N. These findings suggest communication between the two compartments that is generally carbon-based, possibly in the form of carbohydrates and bioactive compounds, such as corticosterone and domoic acid. Nevertheless, some nitrogenous compounds appear to have an endolithic source, indicating a possible contribution of the skeleton to coral animal. Notably, antibiotic treatment greatly increased ^15^N translocation in the tissue but not in the green layer. This highlights an important role of bacteria in nitrogen cycling in the holobiont and in establishing the nitrogen-limiting green layer. Altogether, this study provides the first data about coral skeletal metabolomes. Based on these findings, we propose a model of interactions between coral animal and skeletal bacterial communities, offering a new perspective on the ecological role of endolithic bacteria in corals.

## Introduction

Coral skeletons represent a unique environment for microalgae, bacteria, and fungi, distinct from those in the surface mucus layer and soft tissue [1, 2]. Covered by soft tissue, coral skeletons are generally poorly illuminated and display drastic diurnal fluctuations in oxygen and pH levels [3–6]. This physico-chemical gradient leads to a dense community of endolithic phototrophs in superficial layers of coral skeletons, forming conspicuous green bands (also referred as the green layer) [3, 7] (Box 1). Using isotope tracers, translocation of carbon and nitrogen from the green layer to coral tissue has been documented, suggesting that endolithic microbes in the green layer may constitute an alternative energy and nutrient source for coral holobionts [8–10]. Although this ecological function has historically been attributed to eukaryotic microalgae, studies have detected bacteriochlorophylls and have identified the bacterial *nif*H gene in coral skeletons [11–13]. In the stony corals, *Porites lutea* and *Goniastrea edwardsi*, it was further shown that prokaryotic cells dominate the green layer and exhibit greater carbon fixation activity than do eukaryotic microalgae [14]. These findings suggest that bacterial endoliths may also contribute to the carbon and nitrogen budgets of coral holobionts. Unfortunately, due to the prevalence of eukaryotic microalgae in coral skeletons [15], verification of this interaction between endolithic bacteria and coral animal has been challenging.

**BOX 1:**
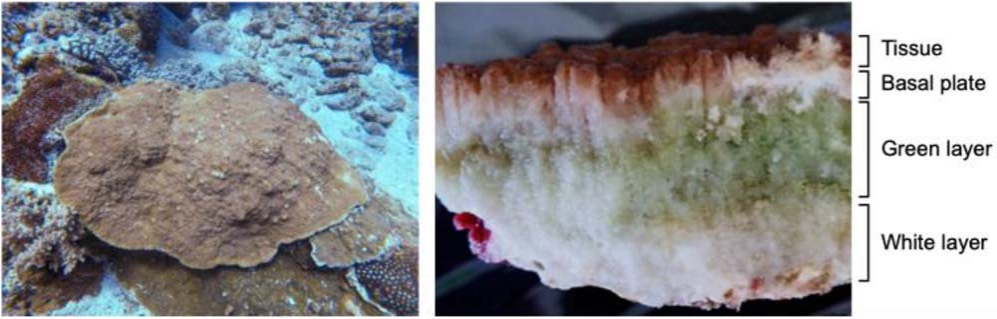
Colored patterns in coral skeletons. The skeletons of stony corals are inhabited by various microorganisms and usually present distinct color bands, which define the skeleton into layers: the covering coral tissue, a thin white basal plate beneath the tissue, a green layer, and a white layer. In most corals, the green layer is colonized by an abundant community of green microalgae from the genus *Ostreobium*. An exception is *I. palifera* from Green Island in Taiwan, of which the green layer is devoid of green microalgae and predominated by green sulfur bacteria (photos are *I. palifera* from Green Island in Taiwan).

Interestingly, in the encrusting coral, *Isopora palifera*, from Green Island in Taiwan, Yang et al. [16] showed the absence of filamentous microalgae or cyanobacteria in the green layer. The endolithic phototrophic community was dominated by anaerobic green sulfur bacteria of the genus *Prosthecochloris* (phylum *Chlorobiota*), indicating an anoxic skeletal environment that is unfavorable to microalgae. This microalga-depleted environment likely extends inward to the skeleton, as reflected by the dominance of anaerobic bacterial classes, *Chlorobia* (17%) and *Chloroflexi* (14%) in the white skeleton (white layer) beneath the green layer, forming a stark contrast to the *Gamma*- and *Alphaproteobacteria*-dominated aerobic bacterial community in the tissue [16]. This skeletal microbiome, devoid or nearly devoid of microalgae, may be attributed to a combination of factors. Specifically, the dense skeleton of *I. palifera* may create a physical barrier to oxygen diffusion, creating an environment unfavorable to microalgae [17, 18]. Geographic location may also be a factor, as the same coral species in the Great Barrier Reef and in Okinawa has abundant microalgal communities in the green layer [2, 19]. Thus, *I. palifera* in Taiwan constitutes a unique model to study the ecological role of endolithic bacteria in coral holobionts. Recently, metagenomic studies have hypothesized metabolic interactions between *I. palifera* and the bacterial community in the green layer [17, 18, 20]. However, evidence for this putative molecular communication has remained nonexistent.

Accordingly, we collected *I. palifera* from the same location as Yang et al. [16] and performed metabolomic analyses on the tissue, green layer, and white layer, to understand metabolism in each. An incubation experiment with stable isotope-labeled inorganic carbon (HCO_3_^−^) and nitrogen (NH_4_^+^) was then conducted to examine metabolite translocation between coral tissue and the skeleton. To investigate the role of tissue-associated bacteria in the putative metabolic crosstalk between coral animal and the endolithic community, antibiotics were also employed in the incubation experiment. This study provides the first metabolomic data for coral skeletons and evidence for metabolic interactions between coral holobionts and bacterial microbiomes in the underlying skeleton.

## Material and methods

### Coral sample collection

Healthy colonies of *I. palifera* were collected at 5–20 m depth near the shore of Green Island (22°40′36.8 N 121°29′34.8 E), Taiwan in June 2020 (*N* = 3) and March 2021 (*N* = 12) for metabolomic analysis and a stable isotope incubation experiment, respectively. Two additional colonies were also collected in May 2025 for a metagenomic analysis to characterize the microbiome in the green layer of this coral.

### Sample preparation for metabolomic analysis

**Figure 1:**
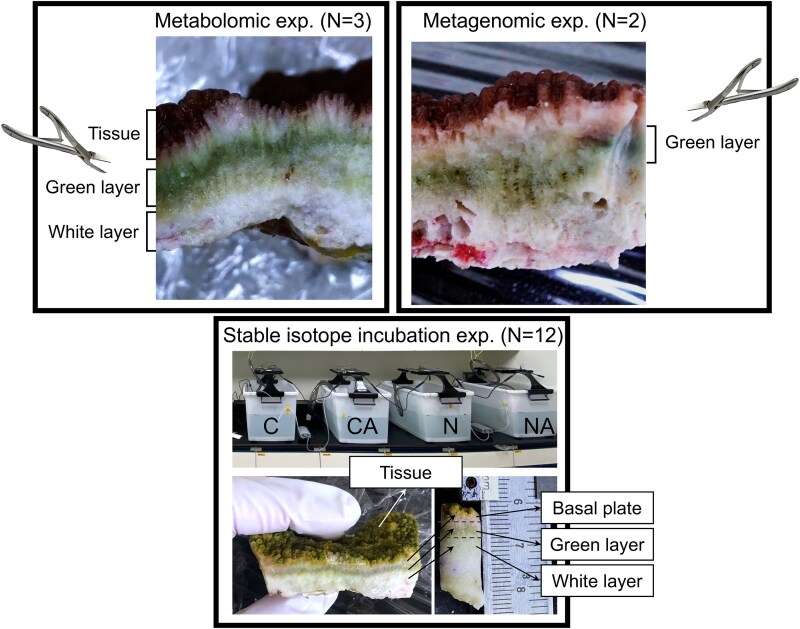
Experimental design of this study. In the metabolomic experiment (left), tissue, the green layer, and the white layer were separated using a bone cutter. In the stable isotope incubation experiment (bottom), colonies of *I. Palifera* were broken into fragments and randomly placed in four tanks for different treatments (C: ^13^CO_3_^2−^, CA: ^13^CO_3_^2−^ + antibiotics, N: ^15^NH_4_^+^, NA: ^15^NH_4_^+^ + antibiotics). Fragments were collected at different times and minicores were drilled for analysis. In the metagenomic experiment (right), the green layer was isolated using a bone cutter.

To preserve the natural metabolomic fingerprint, three healthy *I. palifera* colonies were briefly rinsed with 0.22-μm-filtered artificial seawater (Red Sea Salt, Red Sea, Israel) after sampling and were frozen at −20°C within 1 h. Five replicates were generated from each colony by fragmenting it into subsamples of 10–20 cm^2^ tissue surface area. Soft tissue (with a thin layer of connected skeleton), green layer, and white layer (skeleton beneath the green layer) in each fragment were separated using a bone cutter (Fig. 1). To minimize cross contamination, interfaces between tissue and different skeletal layers were trimmed out with the bone cutter before they were ground using a mortar and pestle. All instruments and equipment were washed with tap water and then with 75% ethanol between samples. Metabolomic analysis was performed by BIOTOOLS Co., Ltd. (New Taipei City, Taiwan) using both gas chromatography-time-of-flight-mass spectrometry (GC-TOF-MS) and ultra-high-performance liquid chromatography tandem mass spectrometry (UHPLC–MS/MS).

### Gas chromatography-time-of-flight-mass spectrometry

For GC-TOF-MS analysis, 50 mg of ground sample were homogenized and dissolved in 450 μL methanol:chloroform (3:1, v/v) and 10 μL L-2-Chlorophenylalanine (1 mg/mL) were added as an internal standard. Samples were centrifuged (12,000 rpm, 4°C, 15 min) and supernatants were extracted. After addition of 5 μL ribose, samples were evaporated to dryness using a vacuum concentrator. Dried extracts were re-dissolved in 50 μL methoxyamine hydrochloride-pyridine solution (20 mg/mL) and incubated at 80°C for 30 min, followed by derivatization with 70 μL BSTFA reagent (1% TMCS, v/v) for 1.5 h at 70°C. Derivatized extracts (1 μL) were then subjected to analysis on an Agilent 7890 gas chromatograph coupled with a time-of-flight mass spectrometer. Gas chromatography was performed in a DB-5MS capillary column in splitless mode at 3 mL/min front inlet purge flow and 1 mL/min gas flow rate in the column, with helium as the carrier gas. The oven was kept at an initial temperature of 50°C for 1 min, raised to 310°C at 10°C/min, and kept at 310°C for 8 min. Mass spectrometry data were acquired in full-scan mode for an m/z range of 50–500 with a solvent delay of 6.25 min. Raw data from the GC-TOF-MS were first processed (including peak extraction, baseline adjustment, deconvolution, alignment, and integration) with Chroma TOF (v4.3x, LECO). Compound annotation was performed by matching mass spectra and retention indices against the LECO-Fiehn Rtx5 database with a cutoff of similarity score > 500.

### Ultra-high-performance liquid chromatography tandem mass spectrometry

For the UHPLC–MS/MS analysis, 15 mg of ground sample was dissolved in 500 μL MeOH:CAN:H_2_O (2:2:1, v/v). As no previous study on a coral skeletal metabolome was available, 0.5 μL L-2-Chlorophenylalanine (1 mg/mL) was added as an internal standard (final concentration: 1 μg/mL) following the default setting suggested by Biotree Biotechnology (Shanghai, China). Extracts were homogenized and incubated at −20°C for 1 h, after which 10 μL of supernatant (centrifuged at 13,000 rpm, 4°C for 15 min) were injected into a Vanquish ultra-high-performance liquid chromatography system coupled with an Orbitrap Elite Mass Spectrometry (Thermo Scientific). Compounds were separated in an Acquity BEH C_18_ column (100 mm × 2.1 mm; 1.7 μm particle; Waters) using a gradient generated with deionized water (solvent A) and acetonitrile (solvent B), both containing 0.1% formic acid. The gradient was initiated for 1 min at 0% solvent B, increased to 100% solvent B in 7 min, held at 100% solvent B for 3 min, and returned to the initial condition in 1 min, followed by re-equilibration for 3 min. The flow rate was 0.25 mL/min. The column oven was set to 40°C. Electrospray ionization (ESI) was employed to ionize column eluents in both positive and negative modes and resulting signals were acquired using data-dependent acquisition mode for an m/z range of 70–1000. Data from UHPLC–MS/MS were processed with XCMS [21] for peak alignment, detection, and integration. MS/MS fragmentation patterns (MS2) were matched against an in-house MS2 database, BiotreeDB (Biotree Biotechnology, China), for compound annotation with an annotation confidence cutoff of MS2 score > 0.5, following the method described in Wan et al. [22].

### Stable isotope incubation experiment

For the stable isotope incubation experiment, collected coral colonies were immediately transferred to the Green Island Marine Research Station near the sampling site. At the research station, colonies were separated into fragments of 10–20 cm^2^ tissue surface area and acclimated overnight in 40-L aquaria filled with sand-filtered natural seawater collected from the ocean close to the sampling site. To investigate translocation of organic carbon (OC) and nitrogen (ON) between coral tissue and the skeleton, 60 fragments (5 fragments ^*^12 colonies) were randomly transferred to four 40-L aquaria with stable isotope-labeled carbon (NaH^13^CO_3_; final concentration = 280 μM) or nitrogen (^15^NH_4_^+^; final concentration = 10 μM). Penicillin (final concentration = 100 units/mL) and streptomycin (final concentration = 0.1 mg/mL) (a broad-spectrum antibiotic cocktail used for tunicate cell culture by Kawamura and Fujiwara [23]), were included to reduce the activity of coral tissue-associated bacteria. The resulting four treatment groups were: (i) NaH^13^CO_3_ only, (ii) NaH^13^CO_3_ plus the antibiotic cocktail, (iii) ^15^NH_4_^+^ only, and (iv) ^15^NH_4_^+^ plus the antibiotic cocktail (Fig. 1). An underwater pump was provided for each aquarium to generate a water current. Incubation was conducted for 24 h at room temperature (~25°C) with a 12-h day-night cycle (320 mmol photons m^−2^ s^−1^; day: 0600–1800; night: 1800–0600). The experiment was initiated at 0700 and three fragments were randomly collected from each treatment group at 0, 3, 6, 12, and 24 h. Health status of coral fragments during the experiment was monitored visually by their gross appearance and by examining their photosynthetic efficiency using a JUNIOR-PAM chlorophyll fluorometer (Heinz Walz GmbH, Germany) at 0 and 24 h. Dark-adapted photosynthetic quantum yields of PS II (F_v_/F_m_) were recorded at the beginning (0 h) and end (24 h) of the experiment for each coral fragment in three technical repeats with a 0.8 s saturating pulse of 4500 mmol photons m^−2^ s^−1^ and a gain value of 12.

### Sample preparation for stable isotope analysis

For each coral fragment collected for the stable isotope incubation experiment, soft tissue was first airbrushed in 15 mL 99% ethanol. Although evidence specifically from *I. palifera* is lacking, ethanol preservation has been reported to alter isotope values and C:N ratios in pearl mussels, with the magnitude positively correlated with preservation duration [24]. To minimize this offset, tissue samples were dried at 50°C for 24–48 h within one week of ethanol preservation and weighed. Vertical mini-cores 9 mm in diameter and 25–40 mm in length were then sampled from skeletons using a diamond-core bit and dried at 50°C for over 48 h (Fig. 1), after which mini-cores were cut with a diamond saw into thin slices (~4 mm) to separate the basal plate, green layer, and white layer (weights of slices were recorded). For tissue samples, 1–2 mg of dried tissue were weighed on tin capsules, combined with 10 μL 1 N HCl to remove inorganic carbon, and dried again at 50°C before packaging for isotope analysis. For skeletal samples, slices of mini-cores were individually dissolved in 1 mL 12 N HCl and concentrated by evaporation at 50°C (to ~300 μL) before being subjected to isotope analysis. Amounts of OC and ON in prepared tissue and skeletal samples were measured with a Flash 2000 Organic Elemental Analyzer coupled with a Delta XP (Thermo Scientific) isotope ratio mass spectrometer (EA-IRMS). OC and ON contents were calculated based on total peak areas of carbon and nitrogen in EA-IRMS, respectively, and normalized by sample weights. Isotope ratios of ^13^C and ^15^N were presented as delta over baseline (Δ), calculated as differences of absolute isotope ratios between those in a sample and references (air for ^15^N and Pee Dee Belemnite (PDB) for ^13^C) using the following equation:


(1) \begin{equation*} \Delta{\left(\frac{{}{}^iE}{{}{}^jE}\right)}_{sample/ reference}=\delta{\left(\frac{{}{}^iE}{{}{}^jE}\right)}_{sample}-\delta{\left(\frac{{}{}^iE}{{}{}^jE}\right)}_{reference} \end{equation*}

where δ(^i^E/^j^E) is the measured δ^13^C or δ^15^N in each sample against standards [25].

### Sample preparation for microbiomic analysis

Coral colonies collected in May 2025 were rinsed with 0.22-μm-filtered artificial seawater and wrapped with plastic wrap before transfer to the laboratory and preservation at −20°C. The green layer was carefully separated using a bone cutter and ground into powder using an iron mortar and pestle (Fig. 1). For each colony, total genomic DNA was extracted from 4 g of ground green layer using DNeasy PowerSoil Pro Kits (QIAGEN, USA) and submitted to Biotools (Taipei, Taiwan) for library construction and metagenome sequencing using an Illumina NovaSeq X system.

### Metabolomic data process and statistical analysis

Metabolomic data were presented as signal intensities of each compound in GC-TOF-MS or UHPLC–MS/MS. For GC-TOF-MS data, integrated peak areas of detected molecules were normalized against the internal standard. For UHPLC–MS/MS data, because the internal standard was not distinguishable from the background signal in either mode (indicating that the amount of internal standard was too low for coral samples), data normalization was conducted based on the total compound peak area in each sample. Following data normalization, GC-TOF-MS data and UHPLC–MS/MS data were combined and replicate samples from the same colony were averaged. A Bray–Curtis dissimilarity matrix was generated and differences in metabolomic profiles among sample origins (tissue, green, and white layers) were examined using a PERMANOVA test (permutation = 999). For individual compounds, normalized abundances were log-transformed and differences between samples were examined using a generalized linear model (GLM) with a Gaussian distribution using the *glm()* function in R. Resulting *p*-values were adjusted for multiple comparisons using the Benjamini–Hochberg false discovery rate (FDR) correction. The threshold for significance was set at *p* < 0.05 for all analyses (FDR < 0.05 for GLM).

### Isotopic data process

For the isotope incubation experiment, data were presented descriptively without statistical analysis. Contents of OC and ON, C:N ratios, and isotope compositions (Δ^13^C and Δ^15^N) were averaged by the depths of skeletal slices (measured as the center of the slice; grouped every 4 mm). Because changes of isotope signals during the experiment involve multiple overlapping biological processes, such as assimilation, dissimilation, and transfer, which are difficult to distinguish precisely, we used the term “translocation” to refer to net changes in isotope signals, regardless of their sources. Considering the high concentrations of isotope tracers employed in the experiment, the putative isotope offset due to ethanol preservation is believed to be negligible, having no effect on data interpretation.

### Metagenomic data process

To verify the dominance of green sulfur bacteria over microalgae in the green layer of *I. palifera*, genome assemblies of *Ostreobium quekettii* (GCA_905146915.1) [26] and *Prosthecochloris* sp. A305 (GCA_004028745.1), the green sulfur bacteria isolated from the same coral [17], were selected for a targeted analysis. Given that the genome assembly of *Prosthecochloris* sp. A305 was only 79.07% complete, genome assembly of another green sulfur bacteria, *Prosthecochloris marina* (GCA_003182595.1; isolated from coastal South China Sea; 99.32% completeness) was also included in this analysis [27], In addition, genome assemblies of the coral *I. palifera* (GCA_964212065.1), *Cladocopium goreaui* (*Symbiodinium* clade C1; available at https://doi.org/10.48610/fba3259) [28], and *Durusdinium trenchii* (*Symbiodinium* clade D; GCA_963970005.1) [29], were included in the analysis to examine intrusion of coral tissue into the green layer. Considering that the *I. palifera* genome assembly was constructed from an Australian population, which differed considerably from our coral in terms of morphology and skeletal microbiome [2], genome assemblies of two phylogenetically closely related corals, *Acropora millepora* (GCA_013753865.1) [30] and *Montipora foliosa* (GCA_036669935.2) [31], were also included to expand the reference pool of stony corals. Illumina sequencing reads were trimmed using Trimmomatic v0.39 [32] and then aligned to these genomes using CoverM with the “bwa-mem2” mapping algorithm [33]. Resulting alignment metrics were determined as total mapped read counts and mean genome coverage.

## Results

### Metabolite profiling

**Figure 2:**
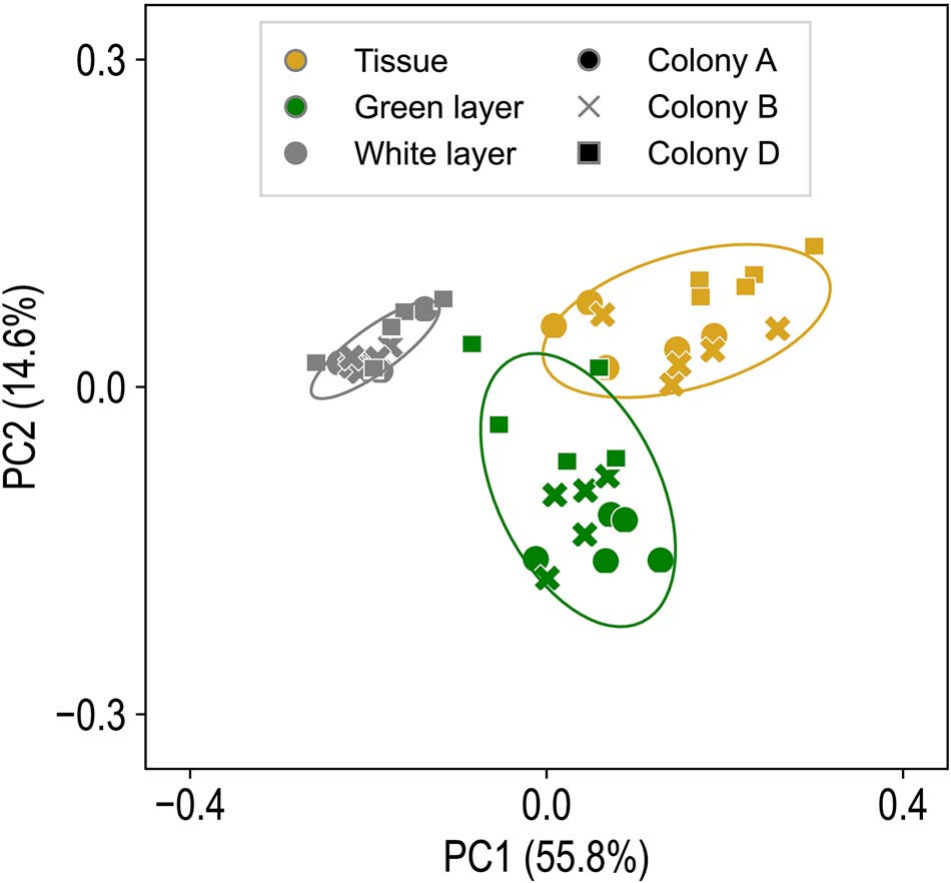
PCoA plot for full metabolomic data (2918 compounds; 45 samples). Significant differences were found among compartments, but not among colonies (*P* < 0.05, PERMANOVA).

From the three coral compartments, i.e. tissue, green layer, and white layer, untargeted metabolomic analysis detected 405 unique features in GC-TOF-MS (internal standard excluded), whereas with UHPLC–MS/MS we detected 2226 and 287 features in positive and negative ion modes, respectively. Principle coordinate analysis (PCoA) showed clear clustering of samples by compartment, with the first two components explaining about 70% of the variation (Fig. 2). Compound annotation yielded a total of 231 compounds with significant confidence (similarity score > 500 for GC-TOF-MS data or MS2 score > 0.5 for UHPLC–MS/MS data, herein, annotated metabolites). Raw data from the metabolomic analysis are provided in Supplementary Material S1.

**Figure 3:**
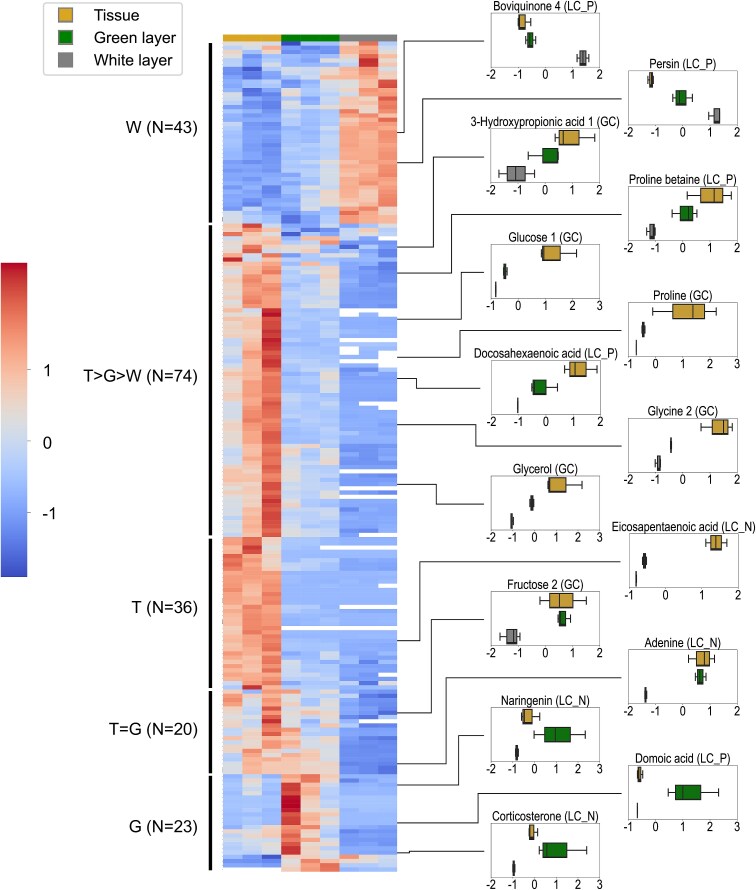
Distributions of metabolites with strong annotation confidence and significant abundance differentiation among compartments (*N* = 196 metabolites). Relative abundances of specific metabolites are highlighted with boxplots on the right, with the *x*-axis representing Z-score transformed GC/LC signals and the *y*-axis representing different sample types.

Among annotated metabolites, 196 showed significant differences in abundance among compartments (GLM, FDR < 0.05; Fig. 3; numerical data are provided in Supplementary Material S1). Approximately two-thirds of annotated metabolites **(*N* = 130) exhibited enrichment in tissue, including several sugars (glucose and tagatose), fatty acids (DHA, EPA, arachidonic acid, proline betaine, and palmitoleic acid), and amino acids (isoleucine, valine, alanine, proline, and glycine). Many lipid derivatives with potential signaling functions were also enriched in tissue, including several lyso-phosphatidylcholines (lysoPCs), lyso-phosphatidylethanolamines (lysoPEs), and monoacylglycerides (MGs). Among these tissue-enriched metabolites, the majority (*N* = 74) showed abundance gradients from tissue to the white layer. Twenty metabolites were shared with considerable enrichment in tissue and the green layer, including sugars (fructose and sorbose), carboxylic acids (3-hydroxypropionic acid), fatty acids (monopalmitin, and pentadecanoic acid), an amino acid (valine), and nucleotides (thymine and adenine). Metabolites specifically enriched in the green layer (*N* = 23) included some energy-related molecules (sucrose and L-palmitoylcarnitine) and several bioactive compounds, e.g. domoic acid, naringenin, corticosterone, androstanol, and brassicasterol. On the contrary, metabolites most highly enriched in the white layer (*N* = 43) included some fungal molecules (persin and boviquinone 4) and many compounds with single or multiple rings, including loquatoside, tyromycic acid, and several catechin or thiazole derivatives.

### Distribution of organic carbon and nitrogen

**Figure 4:**
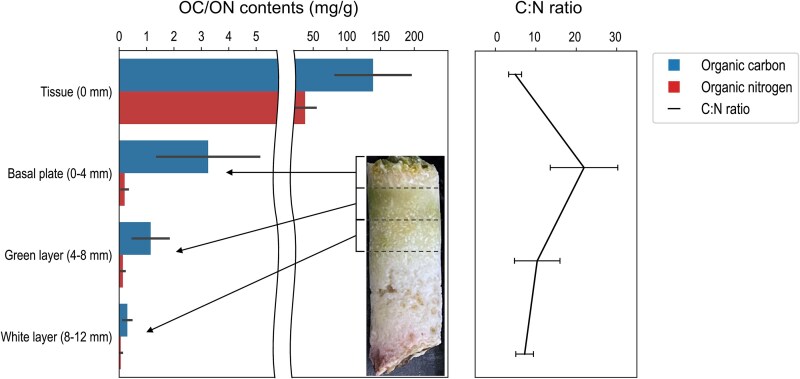
Distributions of OC, ON, and the C:N ratio in *I. Palifera* tissue and skeleton. Data are presented as averages and standard deviations from 3–4 replications (only data from 0 h were used).

All coral fragments were visibly healthy during the stable isotope experiment, with maximum quantum yields (F_v_/F_m_) in the range of 0.60–0.65 before (0 h) and after (24 h) incubation. Both natural OC and ON (measured at 0 h) showed the highest values in tissue (OC: 138.78 ± 55.84 mg/g, ON: 37.91 ± 15.69 mg/g) and declined sharply with depth into the skeleton (Fig. 4). In the most superficial skeletal layer (0–4 mm depth, herein, the basal plate), OC was about 40 times lower (3.25 ± 1.86 mg/g) than in tissue. This value further decreased to 1.15 ± 0.66 mg/g in the green layer (4–8 mm depth), whereas the white layer (8–12 mm depth) showed very low amounts of OC (~0.3 mg/g). In contrast, ON was consistently low in the skeleton (<0.5 mg/g), with values of 0.20 ± 0.12 and 0.13 ± 0.06 mg/g in the basal plate and green layer, respectively, and an even lower value (~0.06 mg/g) in the white layer. Disproportionate declines of OC and ON at 0–4 mm depth resulted in a much higher C:N ratio in the basal plate (21.93 ± 8.35) compared to those in tissue (4.89 ± 1.60) and in the green and white layers (7.07–10.35).

### Stable isotope translocation

**Figure 5:**
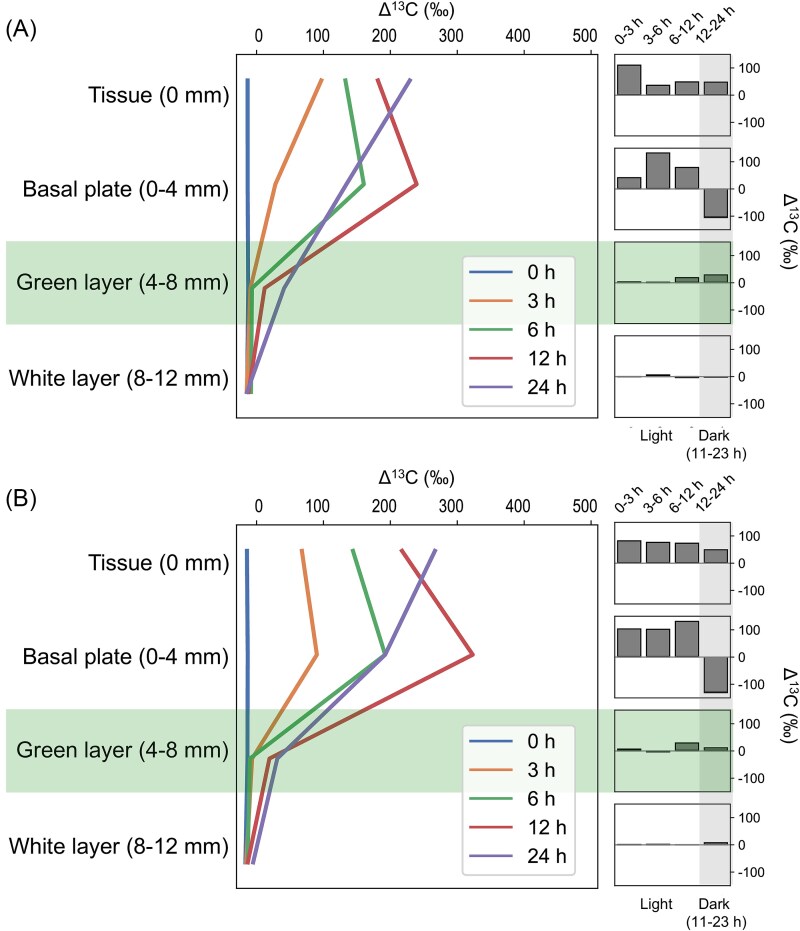
Dynamics of ^13^C translocation in *I. Palifera* tissue and skeleton (left) and corresponding changes at different times (right) without (A) and with antibiotics (B). Data are presented as averages from 3–4 replicates. The green layer and nighttime are highlighted.

**Figure 6:**
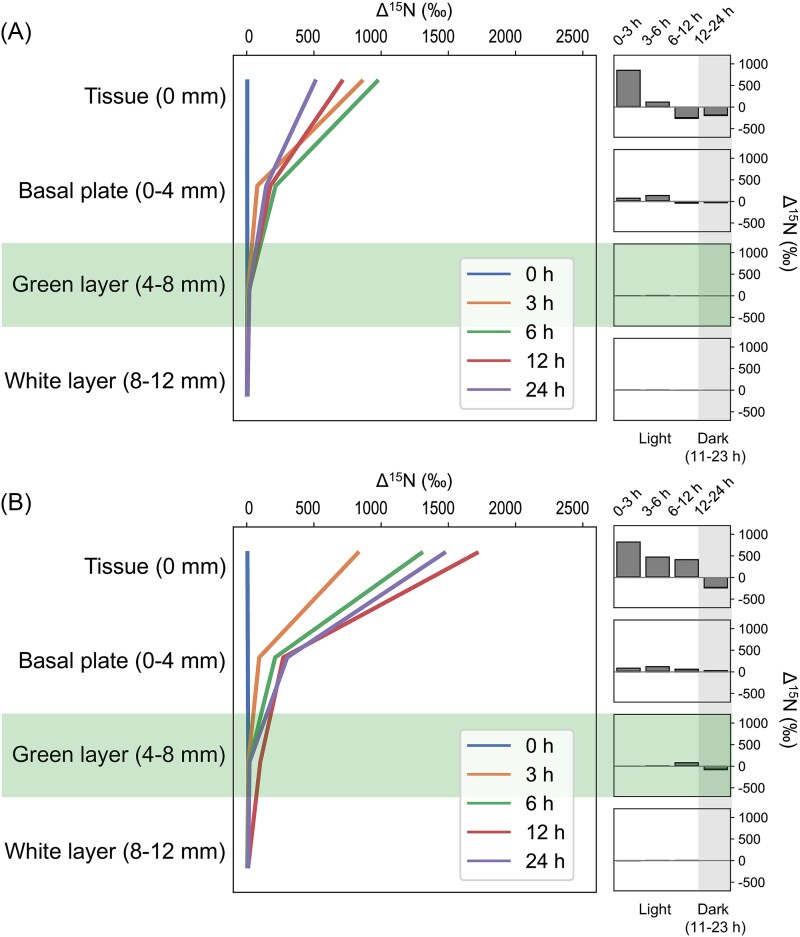
Dynamics of ^15^N translocation in *I. Palifera* tissue and skeleton (left) and corresponding changes at different times (right) without (A) and with antibiotics (B). Data are presented as averages from 3–4 replicates. The green layer and nighttime are highlighted.

During the stable isotope incubation experiment, Δ^13^C increased in tissue and the skeleton (up to the green layer) with time, except for that in the basal plate from 12 h to 24 h (Fig. 5 and Fig. S1). Prominent ^13^C uptake was found in the basal plate at 6–12 h (without antibiotics) and 3–12 h (with antibiotics), when Δ^13^C in the basal plate exceeded that in tissue. Although with relatively weak signals, ^13^C uptake was detectable in the green layer after 12 h. In contrast, increases in Δ^15^N were only found in tissue and the basal plate during the first 6 h (without antibiotics) or 12 h (with antibiotics), after which it decreased with increasing incubation time (Fig. 6 and Fig. S2). In general, addition of antibiotics caused increases in Δ^13^C and Δ^15^N in tissue (+38‰ for Δ^13^C and + 959‰ for Δ^15^N, calculated at 24 h) and the basal plate (+58‰ for Δ^13^C and + 156‰ for Δ^15^N), whereas the effect was less discernible in the green (−10‰ for Δ^13^C and + 1‰ for Δ^15^N) and white layers (+9‰ for Δ^13^C and + 6‰ for Δ^15^N). Raw data from the isotope incubation experiment, including OC, ON, and C:N ratios, are provided in Supplementary Material S2.

### Green layer microbiome

From the green layer of *I. palifera* collected in May 2025, Illumina sequencing yielded a total of 48.5 million reads after processed with Trimmomatic, of which 85.7% successfully mapped to selected reference genomes (Table 1). The majority of sequencing reads mapped to stony corals (37.6%) and Symbiodiniaceae (clade C: 19.2%; clade D: 17.9%), followed by *O. quekettii* (5.2%) and the two *Prosthecochloris* genomes (1.8–4.0%). When genome sizes were considered, stony corals, Symbiodiniaceae, and *O. quekettii* all showed mean coverages lower than 1 (except for *I. palifera* in colony 25 M-C), indicating minor presence of coral tissue and *Ostreobium* microalgae in the green layer. When comparing *Prosthecochloris* and *Ostreobium*, the two green sulfur bacteria were 74–344-fold (*Prosthecochloris* sp. A305) and 20–107-fold (*P. marina*) more abundant than *O. quekettii* in our samples.

**Table 1:** DNA composition of the *I. palifera* green layer. Sequencing reads from colonies 25 M-A and 25 M-C were mapped to 8 reference genomes. Mean coverage was estimated using CoverM.

	Mapped reads (bp)		Mean coverage		Genome size (bp)
	25 M-A	25 M-C	25 M-A	25 M-C	
*Prosthecochloris* sp. A305	268 966	1 689 154	14.3X	106.0X	2 094 032
*Prosthecochloris marina*	138 086	751 557	3.8X	33.0X	2 718 726
*O. quekettii*	1 041 906	1 456 637	0.19X	0.31X	151 902 100
*I. palifera*	3 432 658	8 157 282	0.69X	1.85X	482 731 140
*A. millepora*	1 113 679	1 594 009	0.05X	0.09X	475 381 250
*M. foliosa*	1 641 229	2 271 738	0.04X	0.06X	789 253 060
*C. goreaui*	4 159 508	5 150 750	0.16X	0.16X	1 171 456 500
*D. trenchii*	3 742 606	4 947 179	0.04X	0.06X	1 711 485 200
Unmapped	3 317 820	3 618 116			
Total	18 856 458	29 636 422			

## Discussion

Coral skeletons host diverse endolithic microorganisms that interact metabolically with coral animals [8, 9, 14]. Although recent studies have suggested contributions of bacterial communities in this interaction [14, 18], its verification is challenging due to the prevalence of eukaryotic microalgae in coral skeletons. The green layer of *I. palifera* in Taiwan is devoid of microalgae and is dominated by green sulfur bacteria [16]. Metagenomic data of green sulfur bacteria from the green layer of *I. palifera* have identified functions such as anoxygenic photosynthesis via the rTCA cycle, sulfur metabolism, and nitrogen fixation, suggesting that they may contribute significantly to carbon and nitrogen cycles in the skeleton of *I. palifera* [17, 20]. To confirm this skeletal microbial feature, we collected *I. palifera* colonies from the same location as Yang et al. [16] in 2025. In the green layer of collected colonies, the abundance of green sulfur bacteria of the genus *Prosthecochloris* was 94–452 times higher than that of *Ostreobium* microalgae. This suggests that the green layer microbiome of *I. palifera* in this area is temporally stable and retains the same features as reported by Yang et al. [16]. This coral thus provides a perfect model to study interactions between endolithic bacterial communities and coral animal. In this study we employed this coral to examine translocation of OC and nitrogen between tissue and the underlying skeleton, as well as their corresponding metabolite profiles.

Reflecting the distinct biological components in tissue and different skeletal layers [17], our metabolomic analysis showed clear separation of metabolite profiles in different compartments (Fig. 2). Despite this differentiation, many carbohydrates showed abundance gradients from tissue to the skeleton (Fig. 3). Supporting this metabolite profile, our isotope incubation experiment demonstrated clear translocation of ^13^C from tissue to the green layer (Fig. 5 and Fig. S1). In contrast, translocation of ^15^N was barely detectable in the green layer (Fig. 6 and Fig. S2). This finding is consistent with that in *G. edwardsi* and *P. lutea*, in which ^13^C provided in seawater could be translocated to prokaryotic communities in their green layers, but not ^15^N [14]. Stony corals efficiently cycle nitrogen within holobionts [34, 35]. In contrast, as photosynthetic products from symbiotic algae have very high C:N ratios and are considered “junk food” for corals, stony corals tend to export excess photosynthates to maintain a favorable C:N ratio, for example by mucus excretion or skeletogenesis [36–39]. Secretion of carbon-rich skeletal organic matrix may explain the disproportionate ^13^C and ^15^N translocation in this study and the high C:N ratio in the basal plate of *Isopora*. In coral mucus, metabolism of coral-derived photosynthates by heterotrophic bacteria is accompanied by consumption of organic nitrogen (ON) [40, 41]. The same mechanism may occur in the basal plate of *I. palifera*, creating a barrier for ON inward translocation and facilitating establishment of the nitrogen-limiting green layer. Depletion of ON slows utilization of OC in the basal plate, allowing transportation of remaining OC to the green layer. Thus, our results suggest a potential carbon-based molecular interaction between *I. palifera* and endolithic microbiomes in the green layer. However, this finding differs from that of Sangsawang et al. [9], who found no detectable ^13^C translocation from *P. lutea* tissue to the alga-dominated green layer at a similar isotope tracer concentration (235 μM) and incubation time (24 h). Our result also differs from that in green layer eukaryotic endoliths in *G. edwardsi* and *P. lutea*, in which tissue-to-skeleton translocation of ^13^C and ^15^N were both detected [14]. Therefore, interactions between coral hosts and microbial communities in the green layer may be more complicated and may depend on the taxonomic composition of the microbiome.

**Figure 7:**
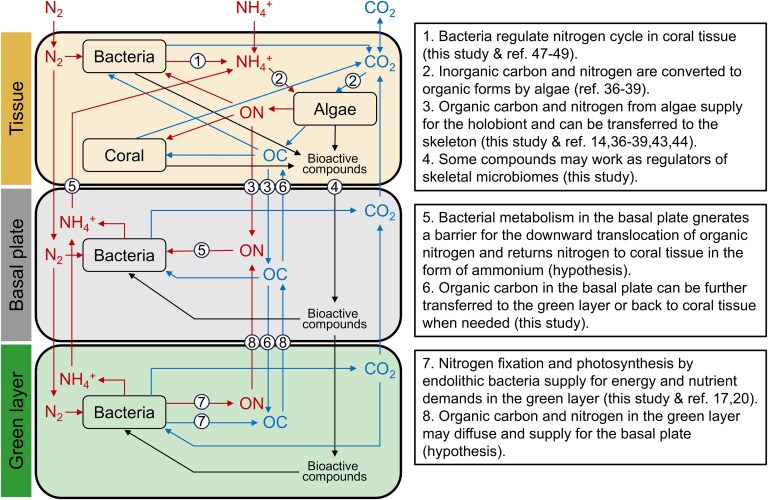
A model for carbon and nitrogen cycles in *I. Palifera* and putative interactions between coral hosts and endolithic microbiomes. Processes involved in the model are highlighted on the side with supporting references. Putative processes are labeled as hypotheses.

In our isotope incubation experiment we also employed antibiotic treatment to examine bacterial contributions to the nutrient translocation process. In *Pocillopora* corals, a 3-day treatment with a similar antibiotic cocktail, consisting of ampicillin (100 μg/mL) and streptomycin (100 μg/mL), significantly altered the microbiomes in coral tissue [42]. Considering the shorter period of our experiment (24 h), we hypothesize that antibiotic treatment functioned by reducing bacterial activity in coral tissue but did not create a microbe-free coral holobiont. Nevertheless, the antibiotic effect is believed negligible on *I. palifera* endoliths, which are non-sensitive to environmental fluctuations due to the dense, non-porous skeleton [19]. Consistent with our hypothesis, the slight increase in ^13^C incorporation in the antibiotic-treated group reflects a reduction of bacterial respiration in coral tissue, which competed with the coral animal for photosynthates from symbiotic zooxanthellae [43, 44]. In contrast to the incorporation of ^13^C, ^15^N incorporation showed a much stronger increase in the antibiotic-treated group. Corals regulate the community of zooxanthellae by limiting nitrogen availability [34, 45, 46]. Although it has long been thought that this function is driven by the coral animal, our results suggest that the bacterial microbiome may contribute significantly to this response. Coral-associated bacteria contribute to the nitrogen cycle by nitrogen fixation, and by nitrifying and denitrifying [47–49]. Disruption of these metabolic activities may perturb nitrogen regulation in the holobiont, leading to overgrowth of symbiotic algae, as observed in corals under ammonium-enriched conditions [50, 51]. Excessive microalgae explain the higher ^15^N incorporation in coral tissue under antibiotic treatment. Importantly, even with the pronounced increase in ^15^N incorporation in tissue under antibiotic treatment, ^15^N uptake in the skeleton was less affected by antibiotic treatment and was merely detectable in the green layer in either group. This finding suggests that inward translocation of organic matter from coral tissue to the skeleton is driven mostly by the coral animal and zooxanthellae. It also supports our hypothesis that the bacterial community in the basal plate consumes ON and establishes the nitrogen-limiting environment in the deeper skeleton. Further studies on microbial community structure are required to corroborate whether antibiotics and disruption of the nitrogen profile affect endolithic bacterial communities in this coral.

Disregarding antibiotic addition, we observed a net increase of Δ^13^C in tissue and a corresponding Δ^13^C decrease in the basal plate from 12 h to 24 h, which roughly corresponds to nighttime in the experiment (Fig. 5). Dynamics of Δ^13^C in coral tissue can be attributed to direct incorporation of ^13^C provided in seawater via photosynthesis or exchange of OC with other compartments, e.g. the mucus layer and skeleton. Our finding suggests that the most superficial skeleton may constitute a reservoir for excess OC, from which it can be retrieved by the coral animal when photosynthesis ceases. Furthermore, our metabolomic analysis identified equivalent enrichment of some carbohydrates in tissue and the green layer, suggesting that these compounds may be synthesized both in coral tissue and the green layer. Thus, bacteria in the green layer may serve as an additional source of OC and may contribute to the energy budget of the holobiont. In addition to carbohydrates, tissue and the green layer shared several nitrogenous compounds, such as some nucleotides and amino acids. Given that inward translocation of ON was negligible, these compounds in the green layer are likely contributed by endolithic bacteria, in which nitrogen fixation activity has been demonstrated [17, 20]. This in situ source of ON explains C:N ratios closer to the Redfield ratio in the skeletal green layer compared to those in the basal plate of our coral. Although the extent is undetermined, we hypothesize that nitrogenous compounds originating in the green layer can be translocated to the basal plate through diffusion, where metabolism generates an extrinsic source of inorganic nitrogen (NH^_4_+^) that supplies the coral holobiont. By drilling and injecting isotope-labeling tracers at specific depths of coral skeletons, Fine and Loya [8] and Sangsawang et al. [9] identified translocation of OC and ON from the green layer to coral tissue. Unfortunately, this strategy is not applicable to our coral as it unavoidably disrupts the anoxic environment in the skeleton. Future development of techniques that can effectively preserve the in situ environment in coral skeletons will be necessary to obtain more reliable evidence for the skeleton-to-tissue metabolic interaction in *I. palifera*.

Interestingly, in our metabolomic analysis we also found specific enrichment of several bioactive compounds in the green layer, including domoic acid, naringenin, and several steroid hormones such as androstenol, corticosterone, and brassicasterol (Fig. 3, Supplementary Material S1). Domoic acid is a neurotoxin generated by red algae and *Pseudo-nitzschia* diatoms, and causes what is commonly known as shellfish poisoning [52, 53]. Although domoic acid stimulates growth of some marine bacteria, such as *Pseudomonas* sp. and *Vibrio* sp. [54], to date there is no documentation of its presence or effects in corals. Naringenin is a plant flavonoid that has antioxidative and antibacterial properties [55, 56]. Although there is no direct evidence for its presence in zooxanthellae, previous studies have detected naringenin in various eukaryotic microalgae [57]. Considering their animal/plant origins, enrichment of these compounds in the green layer may be attributed to active transport from coral tissue to the underlying skeleton, possibly to regulate endolithic microbiomes. Nevertheless, as neither the database of bacterial nor of coral metabolites is comprehensive, their origins and functions in coral skeletons warrant further study.

Based on these findings, we propose a model for interactions between *I. palifera* and endolithic microbiomes in its skeleton (Fig. 7). In this model, organic matter in coral tissue is translocated to the skeleton through diffusion or actively in the form of excess photosynthates. In the basal plate, this organic matter supports metabolism of the endolithic community and generated ammonium is effectively recycled by the coral animal, creating a barrier for ON translocation to the deeper skeleton. In contrast, excess carbon compounds likely penetrate the basal plate and fuel the skeletal microbiome in the green layer. Some bioactive compounds may also be actively transported inward from coral tissue to regulate skeletal microbiomes. In the green layer, nitrogen fixation and photosynthesis by the bacterial community constitute an endolithic source of OC and ON. Although its quantitative contribution is yet undetermined, organic matter generated in the green layer may be transported outward, contributing to the C and N budgets of the holobiont. This model thus suggests active interactions between coral animal and endolithic bacteria and an ecological role of green layer bacterial communities as an additional carbon and nitrogen source for holobionts.

## Supplementary Material

FigS1_v2_ycaf192

FigS2_v2_ycaf192

Supplementary_material_1_v3_ycaf192

Supplementary_material_2_v3_ycaf192

## Data Availability

Metabolomic and isotopic data generated or analyzed during this study are included in this published article and the supplementary information files. Metagenomic data generated in this study are available at NCBI under BioProject PRJNA1300824.
