# Supplementary figures and images for: Metabolic interactions between coral animal and endolithic bacterial communities
Source: ISME Commun. 2024 Oct 28;5(1):ycaf193. doi: 10.1093/ismeco/ycaf193 (PMC12624861; doi:10.1093/ismeco/ycaf193)

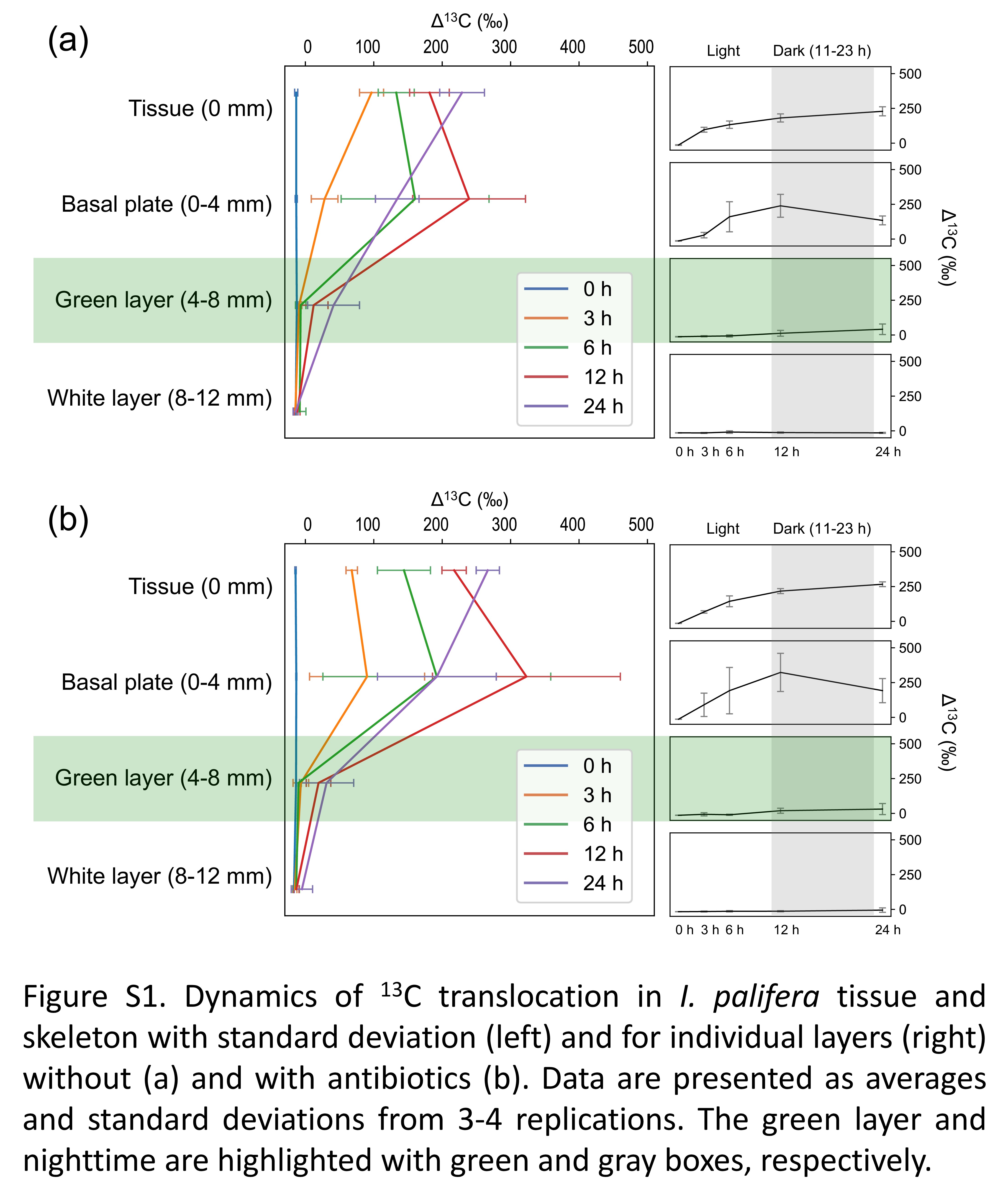

Supplement: FigS1_v2_ycaf192 [file figs1_v2_ycaf192.jpeg]

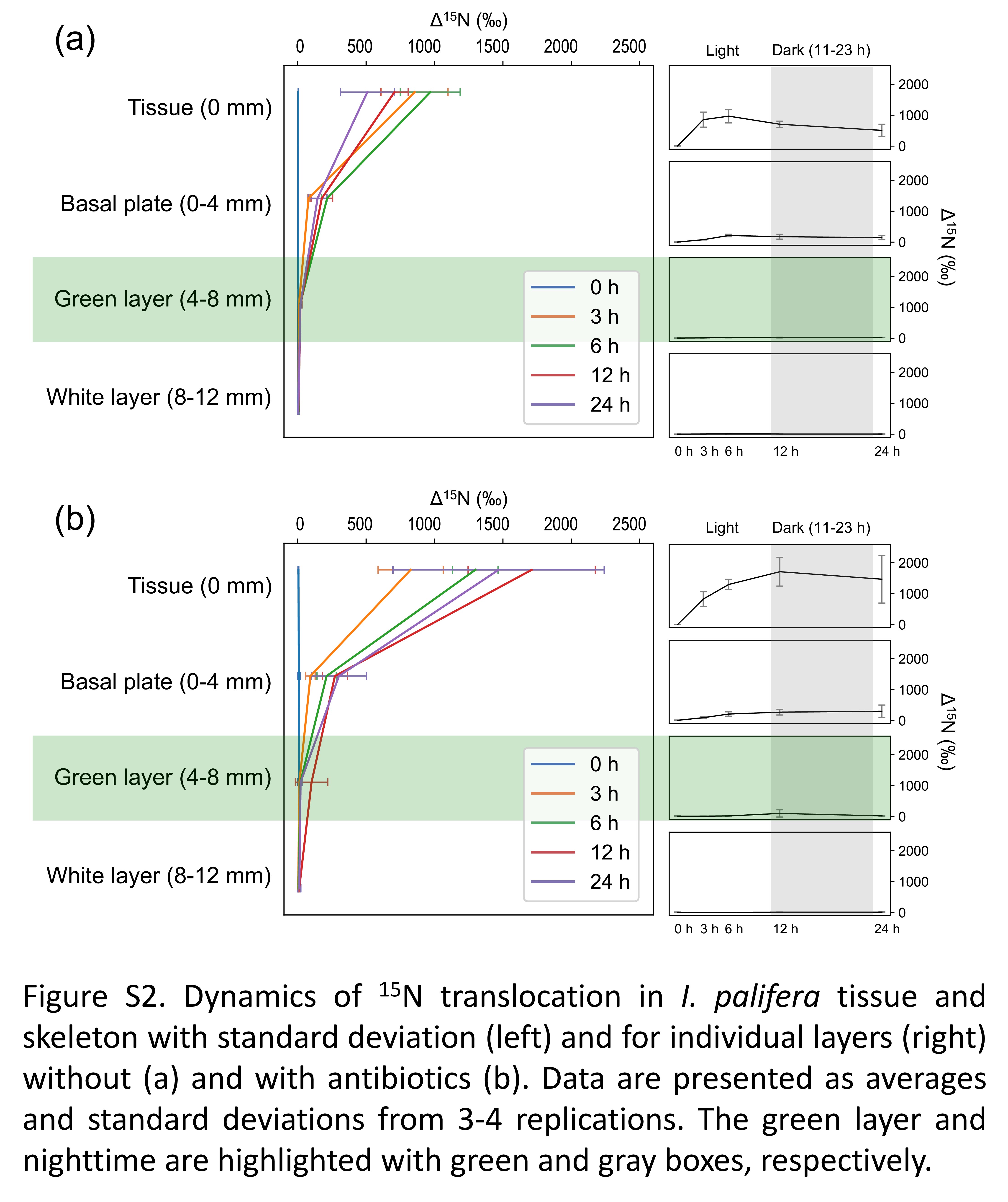

Supplement: FigS2_v2_ycaf192 [file figs2_v2_ycaf192.jpeg]
